# Supplementary material for: Does Self-Reassurance Reduce Neural and Self-Report Reactivity to Negative Life Events?
Source: Front Psychol. 2021 Sep 28;12:658118. doi: 10.3389/fpsyg.2021.658118 (PMC8505763; doi:10.3389/fpsyg.2021.658118)
Supplement: Supplementary file 1 [file Table_1.DOCX]

**TABLES**

| Area | Cluster peak  co-ordinates (XYZ) | Peak  t-score | K_E_ | P_FWE-corr_ |
| --- | --- | --- | --- | --- |
| *Reassurance: Emotional – Neutral* | | | | |
| Left Angular Gyrus  Left Inferior Parietal | -40 -56 36 | 5.53 | 244 | .001 |
| Left Lingual Gyrus  Left Calcarine  Right Lingual  Right Lingual  Right Calcarine  Right Cerebellum  Left Frontal Supp Medial  Right Frontal Supp Medial | -10 -82 4  12 -80 4  2 50 34 | 5.23  5.07  4.44 | 305  299  147 | .001  .001  .018 |

***Table 1.*** Brain regions that showed significantly greater activation for self-reassurance (emotion - neutral). Peak thresholded at FWE, *p* <0.05 with K = 144 and coordinates reported in MNI-space. Cluster labelling was used from the AAL toolbox implemented in SPM12.

| Area | Cluster peak  co-ordinates (XYZ) | Peak  t-score | K_E_ | P_FWE-corr_ |
| --- | --- | --- | --- | --- |
| *Criticism: Emotional – Neutral* | | | | |
| Left Calcarine  Right Lingual | -4 -52 36 | 9.21 | 11295 | .001 |
| Left Lingual  Left Precuneus  Right Calcarine  Left Fusiform  Left Cuneus  Left Middle Cingulate  Right Cuneus  Left Inferior Occipital  Right Fusiform  Right Precuneus  Right Posterior Cingulate  Left Cerebellum  Right Cerebellum  Vermis  Right Middle Cingulate  Right Posterior Cingulate  Left Inferior Temporal  Vermis  Left Crusl 1 Cerebellum  Left Supplementary Motor Area  Left Parahippocampal  Left Hippocampus  Left paracentral Lobule  Right Inferior Occipital  Right ParaHippocampal  Right Cerebellum Crusl 1  Left Middle Temporal  Right Cerebellum 3  Right Middle Cingulate  Left Putamen  Left Insula  Left Inferior Orbital  Left Frontal Inferior Tri  Left Hippocampus  Left OFCpost  Left Amygdala  Left Inferior Temporal  Right Caudate  Right Insula  Right Frontal Inferior Oper  Right Putamen  R Rolandic Operculum  Left Frontal Superior Medial  Right Frontal Superior Medial  Left Caudate  Left Thalamus Putamen  Left Thalamus LP  Left Angular  Left SupraMarginal  Left Mid Occipital  Left Mid Temporal  Left Sup Temporal  Left Inferior Parietal  Left Precentral  Left Postcentral  Left Parietal Sup  Left Paracentral Lobule | -28 19 -14  10 0 22?  -4 46 36  -8 -18 22  -44 -46 22  -30 -28 48 | 7.45  6.02  5.72  5.62  5.45  5.26 | 677  349  145  350  494  625 | .001  .001  .026  .001  .001  .001 |

***Table 2.*** Brain regions that showed significantly greater activation for self-criticism (emotion - neutral). Peak thresholded at FWE, *p* <0.05 with K = 144 and coordinates reported in MNI-space. Cluster labelling was used from the AAL toolbox implemented in SPM12.

| Area | Cluster peak  co-ordinates (XYZ) | Peak  t-score | K_E_ | P_FWE-corr_ |
| --- | --- | --- | --- | --- |
| *Criticism (Emotional – Neutral) – Reassurance (Emotional – Neutral):* | | | | |
| Left Putamen  Left Hippocampus  Left Pallidum  Left Insula  Right Hippocampus  Left Thalamus VL  Left Caudate  Left Thal VPL  Left Thal LP  Left Precuneus  Left Calcarine  Left Lingual  Vermis_4_5  Left Posterior Cingulate  Left Cerebellum  Left Cuneus  Left Inferior Occipital  Left Middle Occipital  Left Middle Cingulate  Right Middle Cingulate  Left Supp Motor Area  R Supp Motor Area  Left Middle Occipital  Left Angular  Left Middle Temporal | -36 -16 -10  24 -24 18  -12 -12 18  -4 -56 4  -28 -84 -4  0 14 36  -38 -72 24 | 5.33  4.49  4.46  256  117  115  127 | 314  147  158  256 | .001  .001  .001  .001  .001  .001  .001 |

***Table 3.*** Brain regions that showed significantly greater activation for self-criticism (emotion - neutral) - self-reassurance (emotion - neutral). Peak thresholded at FWE, *p* <0.05 with K = 110 and coordinates reported in MNI-space. Cluster labelling was used from the AAL toolbox implemented in SPM12.
